# Toward brain-computer interface speller with movement-related cortical potentials as control signals

**DOI:** 10.3389/fnhum.2025.1539081

**Published:** 2025-04-02

**Authors:** José Jesús Hernández-Gloria, Andres Jaramillo-Gonzalez, Andrej M. Savić, Natalie Mrachacz-Kersting

**Affiliations:** ^1^Laboratory for Biomedical Microtechnology, Department of Microsystems Engineering-IMTEK, University of Freiburg, Freiburg, Germany; ^2^Institute of Sport and Sport Science, Albert-Ludwigs-Universität Freiburg, Freiburg, Germany; ^3^Science and Research Centre, University of Belgrade – School of Electrical Engineering, Belgrade, Serbia; ^4^BrainLinks-BrainTools Center, IMBIT, Albert-Ludwigs University of Freiburg, Freiburg, Germany

**Keywords:** movement-related cortical potentials, brain-computer interface speller, control signal, amyotrophic lateral sclerosis, electroencephalography

## Abstract

Brain Computer Interface spellers offer a promising alternative for individuals with Amyotrophic Lateral Sclerosis (ALS) by facilitating communication without relying on muscle activity. This study assessed the feasibility of using movement related cortical potentials (MRCPs) as a control signal for a Brain-Computer Interface speller in an offline setting. Unlike motor imagery-based BCIs, this study focused on executed movements. Fifteen healthy subjects performed three spelling tasks that involved choosing specific letters displayed on a computer screen by performing a ballistic dorsiflexion of the dominant foot. Electroencephalographic signals were recorded from 10 sites centered around Cz. Three conditions were tested to evaluate MRCP performance under varying task demands: a control condition using repeated selections of the letter “O” to isolate movement-related brain activity; a phrase spelling condition with structured text (“HELLO IM FINE”) to simulate a meaningful spelling task with moderate cognitive load; and a random condition using a randomized sequence of letters to introduce higher task complexity by removing linguistic or semantic context. The success rate, defined as the presence of an MRCP, was manually determined. It was approximately 69% for both the control and phrase conditions, with a slight decrease in the random condition, likely due to increased task complexity. Significant differences in MRCP features were observed between conditions with Laplacian filtering, whereas no significant differences were found in single-site Cz recordings. These results contribute to the development of MRCP-based BCI spellers by demonstrating their feasibility in a spelling task. However, further research is required to implement and validate real-time applications.

## 1 Introduction

In recent decades, the field of Brain Computer Interface (BCI) technology has undergone remarkable advancements, transitioning from theoretical concepts to practical applications (Aricò et al., 2020; Xu et al., 2024). A BCI is defined as a system in which commands are sent to external devices using only brain signals and without passing through the brain’s normal output pathways (Wolpaw et al., 2002). This innovative technology holds significant potential to empower individuals with motor disabilities, such as patients with Amyotrophic lateral sclerosis (ALS) (Masrori and Van Damme, 2020), as it could provide them with a means to communicate and engage with the world.

The basic structure of a BCI includes signal acquisition, feature extraction, classification or feature translation, and output or command (Cecotti, 2011; Shih et al., 2012; Wolpaw et al., 2002). During signal acquisition, brain signals are collected and used to extract specific features that are translated into commands capable of operating a device through mathematical algorithms (Shih et al., 2012; Wolpaw et al., 2002).

The efficacy of a BCI is linked to technological progress and understanding the basic underlying neuroscience, as well as the interaction between the user and the system. The features extracted are commonly individualized for the specific user to allow accurate and efficient translation into device commands (Abiri et al., 2019; Wolpaw et al., 2002; Xu et al., 2024).

One promising application of BCI technology is the development of “BCI Spellers, “which have gained substantial attention for their potential to allow communication for individuals with loss of voluntary motor control due to neurological conditions (Chaudhary et al., 2021; Rezeika et al., 2018).

BCI speller systems fall into two main acquisition method groups: invasive and non-invasive. Invasive methods involve surgical procedures to implant electrodes, providing high spatial and temporal resolution but requiring a potentially risky procedure, making them a less approachable solution (Chaudhary et al., 2022; Oh et al., 2024; Xu et al., 2024). On the other hand, non-invasive methods acquire signals from the scalp which introduces a loss in signal quality though it makes them easier to use and more user friendly (Abiri et al., 2019; Oh et al., 2024; Xu et al., 2024).

Traditional non-invasive BCI spellers and virtual menus rely on control signals such as the P300, steady-state visually evoked potentials (SSVEPs), and event-related spectral perturbations (ERSPs) (Abiri et al., 2019; Cecotti, 2011; Gannouni et al., 2017; Rezeika et al., 2018; Savić et al., 2013). These paradigms have achieved considerable success, with P300 and SSVEP-based BCIs demonstrating high accuracy and generally requiring minimal user training. However, the performance of P300 and SSVEP BCIs can be influenced by factors like stimulus presentation and individual user characteristics. ERSPs, while offering a different approach, can involve more extensive user training. These factors are important considerations when evaluating the suitability of different BCI paradigms for specific applications (Table 1).

**Table 1:** Advantages and disadvantages of brain signals for BCI-spellers and menus.

Brain signals	Advantages	Disadvantages
P300 and SSVEP	Requires minimal user training for control signal induction (Nicolas-Alonso and Gomez-Gil, 2012; Rezeika et al., 2018) High bit rate [SSVEP: 30–300 bits/min. P300: 20–80 bits/min (Li et al., 2021; Rezeika et al., 2018)] Limited number of EEG channels required(Li et al., 2021; Nicolas-Alonso and Gomez-Gil, 2012) Multiclass control (Rezeika et al., 2018)	Requires permanent attention to external visual stimuli (Abiri et al., 2019; Li et al., 2021; Rezeika et al., 2018) Decrease in performance due to habituation may occur (Nicolas-Alonso and Gomez-Gil, 2012) May cause visual fatigue in some users (Abiri et al., 2019; Kundu and Ari, 2022; Rezeika et al., 2018)
ERSP	Stimulus independent control signal induction (Nicolas-Alonso and Gomez-Gil, 2012; Rezeika et al., 2018) May enable multiclass control using different motor imagery tasks (Nicolas-Alonso and Gomez-Gil, 2012; Rezeika et al., 2018) Useful for users with visual impairments (Nicolas-Alonso and Gomez-Gil, 2012; Rezeika et al., 2018)	Variability in subject specific signal features implying the need for extensive user training and system calibration (Abiri et al., 2019; Nicolas-Alonso and Gomez-Gil, 2012; Rezeika et al., 2018) Not all users are able to obtain control (Nicolas-Alonso and Gomez-Gil, 2012) Multichannel EEG recordings are required for multiclass control (Abiri et al., 2019; Nicolas-Alonso and Gomez-Gil, 2012) Lower bit rate (3–35 bits/min) (Nicolas-Alonso and Gomez-Gil, 2012; Rezeika et al., 2018) Extended user training periods (Kundu and Ari, 2022; Rezeika et al., 2018)

In this context, movement-related cortical potentials (MRCPs) as a control signal may provide a feasible alternative to counteract some of the aforementioned limitations. MRCPs are low-frequency negative shifts in the EEG that occur approximately 2 s before the initiation of voluntary movement and can also be induced through motor imagery (Shakeel et al., 2015; Shibasaki and Hallett, 2006; Wright et al., 2011). The MRCP consists of various components associated with motor preparation and execution, including the Bereitschaftspotential (BP), an early negative shift, the negative slope (0.5 prior to movement onset) and motor potential (Crammond and Kalaska, 2000; Jochumsen et al., 2017; Shibasaki and Hallett, 2006).

ERSPs accompany voluntary movements and motor imagery as well but can also be induced or modulated by various cognitive tasks or affected by parallel streams of sensory inputs or the user’s mental state (Savić et al., 2020). Moreover, ERSPs exhibit variability in their patterns among individuals, in terms of frequency bands of interest and spatio-temporal dynamics. As a result, extensive user training and calibration of both subjects and the BCI are required to operate the system using motor imagery-induced ERSPs (Savić et al., 2020).

In contrast, MRCPs are direct and reproducible signatures of motor preparation, execution, or imagery, exhibit low user training requirements, are adaptable through training and detectable before movement onset (Jochumsen et al., 2018; Savić et al., 2020, 2021; Wright et al., 2011, 2012). However, due to their low signal-to-noise ratio, they have been less commonly used for BCI control. Additionally, MRCPs have shown particular sensitivity to specific visual cues, which can disrupt their morphological features and may limit their use in cue-based applications such as menus and spelling (Savić et al., 2014).

Despite this, recent studies have demonstrated the concept and feasibility of single-trial MRCP detection using novel feature extraction and machine learning techniques (Jiang et al., 2015; Savić et al., 2021). These advances in MRCP detection and classification for BCI control purposes, they have not yet been specifically applied to BCI spelling applications.

This study investigates the feasibility of using MRCPs as control signals for a BCI speller system. While other speller approaches are mentioned as reference points, a direct comparison goes beyond the scope of this study. Our primary goal is to evaluate whether MRCPs can be effectively utilized in this specific framework. Unlike motor imagery-based BCIs, we examine MRCPs elicited by executed movements, where participants actively performed dorsiflexion. To establish a foundational understanding of MRCP behavior in this context, we conducted the experiments in an offline setting, collecting and analyzing MRCP data *post-hoc* without real-time detection. Instead of implementing machine learning classification or automated MRCP detection, we established predefined criteria to assess the presence of MRCPs, evaluating their reliability as the success rate. Additionally, we explored how MRCP features are influenced by the cognitive load, visual cues, and stimuli imposed by the spelling task.

## 2 Materials and methods

### 2.1 Participants

Fifteen healthy young adults (seven females, eight males; mean age = 24.38 ± 2.98 years) without any neurological disorders or other impairments voluntarily participated in this study. Due to a technical issue with the EEG system during data acquisition, specifically the failure of a EEG or EMG electrode, data from two subjects were excluded from further analysis. This malfunction resulted in unusable data for those channels, rendering the entire dataset for those participants unusable. This exclusion was based on the objective criterion of complete electrode failure. Consequently, the final dataset comprised 13 participants (seven females, six males). All participants provided informed consent prior to their inclusion in the experiment. The study protocol was conducted according to the Declaration of Helsinki.

### 2.2 Experimental setup

**Figure 1:**
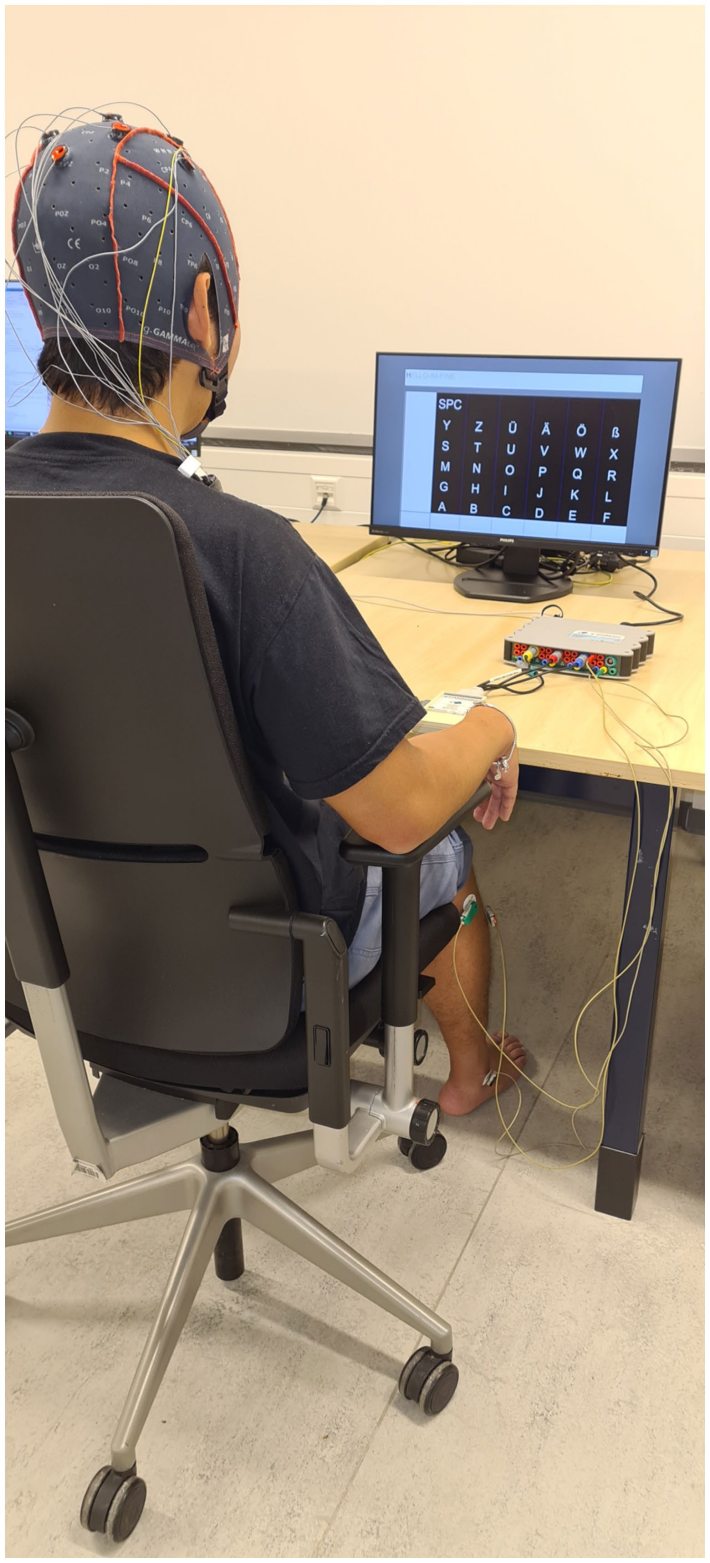
The experimental set up for the experiment with the keyboard on the screen and EEG and EMG electrodes connected to the g.USBamp system.

During all sessions, monopolar EEG signals were recorded using an active EEG electrode system (g.GAMMAcap; g.tec Medical Engineering, GmbH, Austria) connected to a g.USBamp amplifier (g.tec Medical Engineering, GmbH, Austria). Ten electrodes were placed over the motor cortex according to the standard international 10–20 system: FP1, Fz, FC1, FC2, C3, Cz, C4, CP1, CP2, and Pz. The ground electrode was placed centrally on the frontopolar region (FPz), and the contralateral earlobe was used for reference. Surface EMG electrodes were placed on the musculus tibialis anterior (TA) to determine movement onset (Figure 1). All data were sampled at a rate of 1,200 Hz.

**Figure 2:**
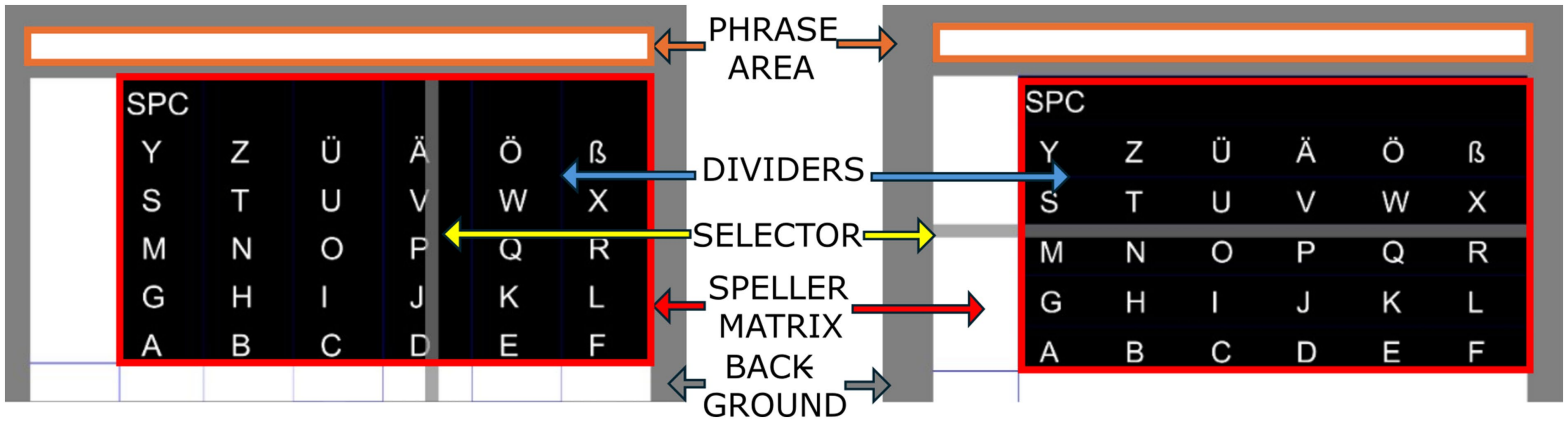
The speller with custom designed interface. The grey bar is the moving selector and phrase area is where chosen characters would appear. The selector first scanned the speller matrix from left to right to identify the column containing the target letters, predefined by the condition (control, phrase spelling, or random). Once the column was reached, the selector changed direction and scanned from bottom to top to locate the target letter’s row. After selecting the row, the selector restarted its position to search for the next target letter, following the same column-first, then row-selection process.

During the actual experiment, subjects interacted with a custom-made speller interface consisting of a 6 × 6 matrix design (Farwell and Donchin, 1988) with characters to choose from and a continuously moving selector (Figure 2). The selector consists of a gray bar that moves horizontally (x-axis) until a selection is made, then changes direction to move vertically (y-axis).

The BCI speller operates as a simulation, where letter selections were predefined, and the system automatically pauses at the corresponding column or row, assuming that the subject would initiate movement during the designated time. Each column and row selection lasted for 2 s. After each selection, the bar paused for 2 s before resuming movement for the next selection. The speller includes a blank area at the top displaying the selected letters or a predefined phrase, with the last selected letter highlighted.

For each condition, the target phrase was presented at the beginning of the experiment in the Phrase Area of the speller interface (Figure 2). The selector moved from left to right, sliding over the columns (which were internally divided within the speller matrix). Once the selector reaches the end of the predefined column of the target letter, it automatically changed direction and moved from bottom to top to stop at the end of the corresponding row. The rows and columns were visually marked with dividers to aid user orientation (Figure 2). Once a letter was selected the bar reset its position at the left of the screen to restart the sliding. Participants were cued to execute a movement when the selector reached the target location (column/row) of the letter. This ensured that MRCPs were aligned with voluntary movement at the desired letter.

### 2.3 Experimental procedures

All measurements were conducted at the neurophysiological laboratory of the Department of Sports and Sports Science of the University Freiburg.

The subjects were instructed to perform a ballistic dorsiflexion of the dominant leg, executing the movement when the bar was around the midpoint of the column or row containing the desired character. Each subject completed three different phrases, each consisting of 13 characters, resulting in 26 trials or 26 possible MRCPs per phrase (Table 2).

**Table 2:** The conditions used in determining the use of MRCPs for BCIs.

Condition	Description
Letter or control condition	The control condition, consisting of repetitive “O” characters, was designed to minimize cognitive load by requiring simple, repetitive motor execution with minimal cognitive demands related to letter selection or memory.
Phrase spelling condition	Copy spelling condition in which the subject simulates the spelling of the phrase “HELLO IM FINE.” This condition aims to study the MRCP during the spelling task condition. This condition required participants to recall the sequence of letters, and coordinate motor actions with the spelling task.
Random condition	A phrase of the same length as the copy spelling phrase was generated by randomizing its characters. This randomized phrase was presented to the subjects prior to the experiment three. This condition required participants to process and select letters without the aid of semantic or contextual cues, potentially increasing demands on attention and working memory.

To evaluate MRCP performance in conjunction with a simulated spelling task, three conditions were tested. These conditions were designed to assess how MRCPs are affected by the varying demands of a spelling task, ranging from minimal cognitive load to more complex scenarios involving memory and letter selection. Cognitive load, in this study, refers to the mental effort imposed on participants by the task demands, including factors such as memory, attention, and decision-making. The three conditions were: a control condition using repeated selections of the letter “O” to isolate movement-related brain activity with minimal cognitive load; a phrase spelling condition with structured text (“HELLO IM FINE”) to simulate a meaningful spelling task with moderate cognitive load, reflecting the memory and planning involved in spelling a familiar phrase; and a random condition using a randomized sequence of letters to introduce higher task complexity, as participants needed to process and select letters without the aid of linguistic or semantic context, thus increasing demands on attention and working memory.

### 2.4 Data processing and statistical analysis

All signal analysis was done with a custom MATLAB script and GUI (version 2022a, MathWorks, Natick, USA). The CZ electrode was chosen for the subsequent analysis, as it is the electrode that is highly associated with lower limb movement (Do Nascimento et al., 2006; Shibasaki et al., 1986; Shibasaki and Hallett, 2006). The EMG data was used as supplementary data for the detection of the movement and as part of the criteria for selecting the MRCPs.

**Figure 3:**
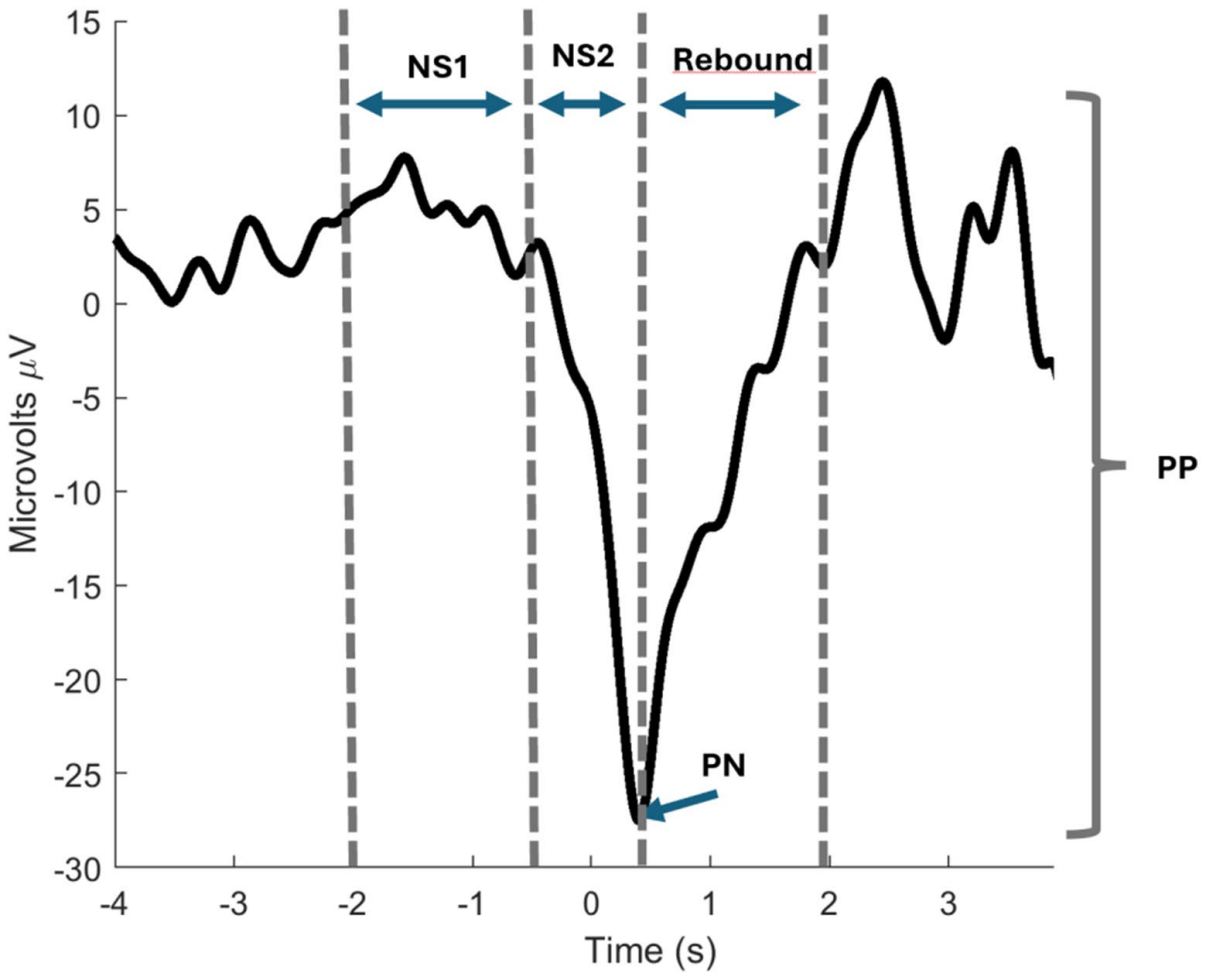
MRCP features. The black trace represents an example of an MRCP, with dotted lines indicating the time intervals used to compute the slopes (NS1, NS2, and rebound). The PN (Peak Negativity) is identified as the minimum value on the trace, while the PP (Peak-to-Peak) represents the overall amplitude of the MRCP (the difference between the minimum and maximum value of the signal from 2 s before PN to 2 s after).

**Figure 4:**
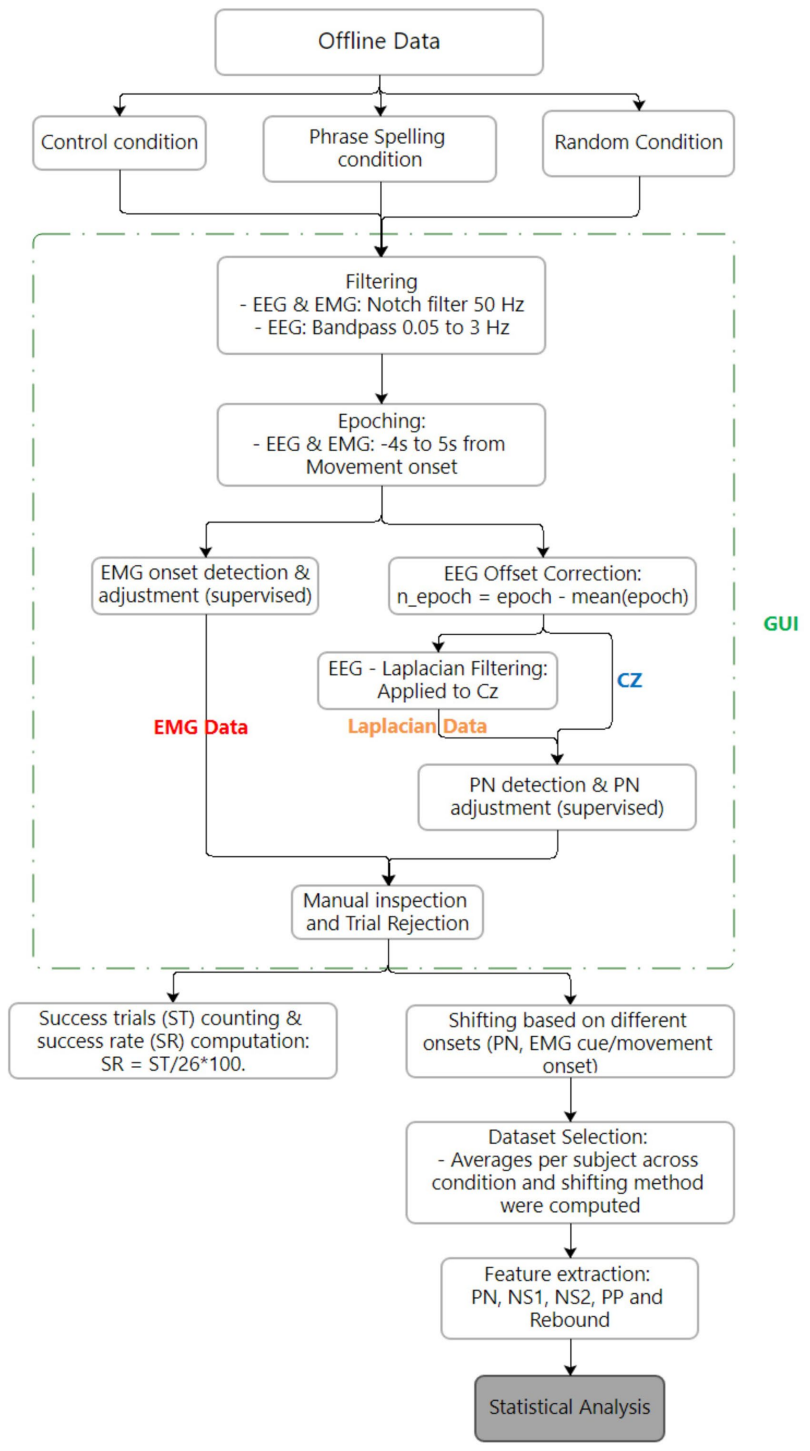
Preprocessing block for MRCP signals.

**Figure 5:**
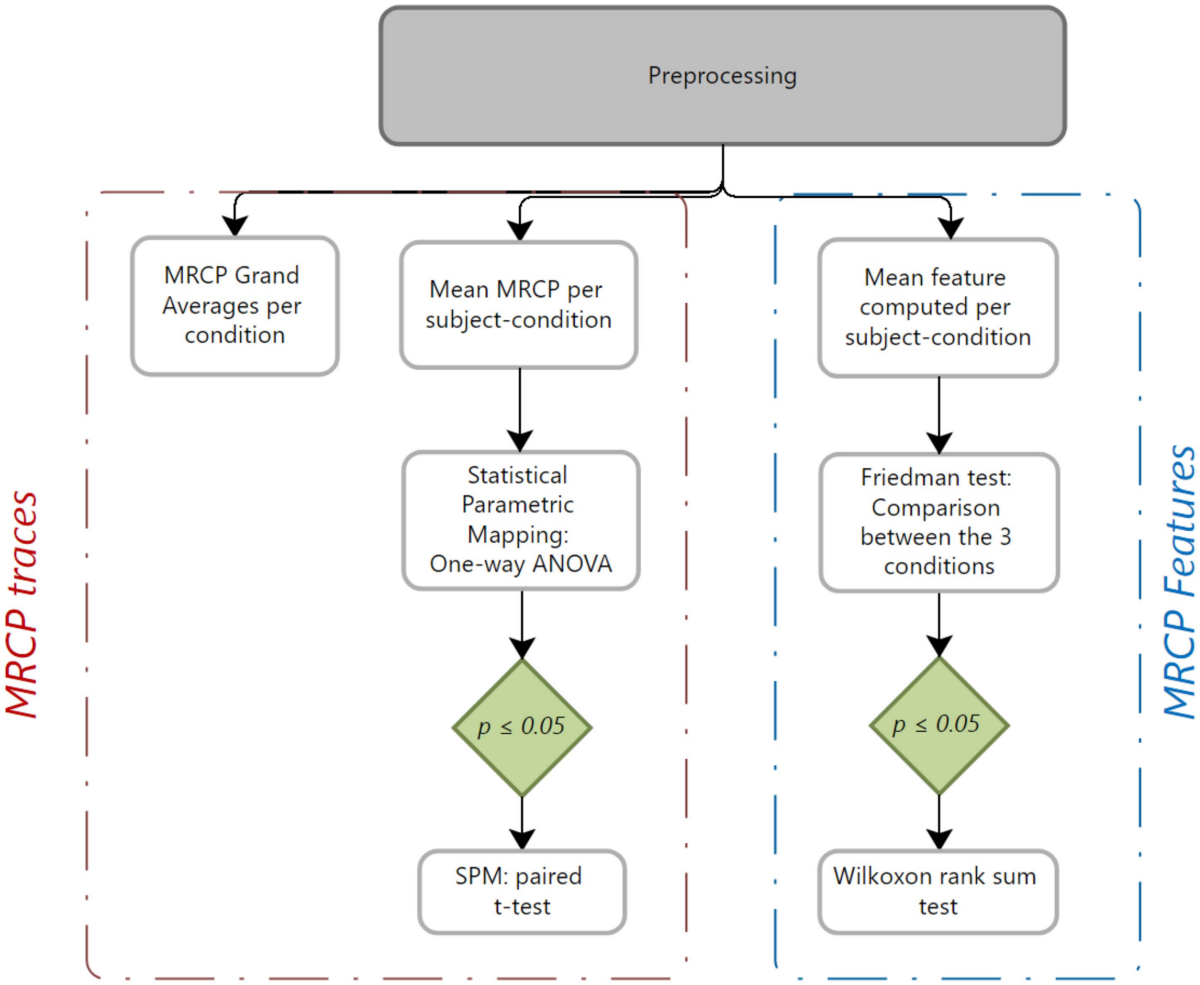
Statistical analysis block for MRCPs and its specific features.

**Figure 6:**
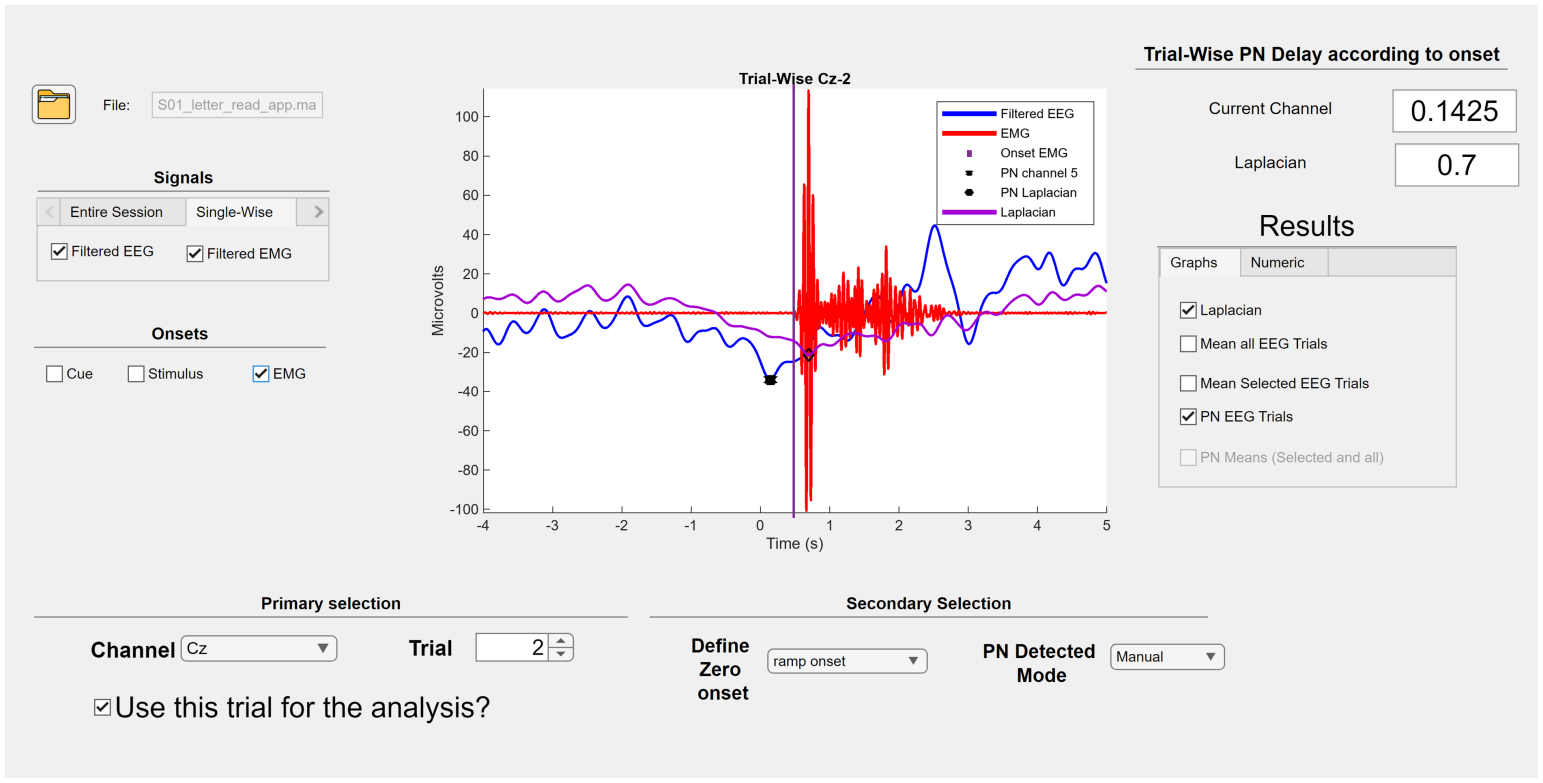
MATLAB custom made GUI used for the preprocessing, segmentation, and movement verification.

A simplified signal processing pipeline was implemented, avoiding computationally expensive techniques such as Independent Component Analysis (ICA) for artifact detection. Instead, we employed filtering and epoch-based processing techniques that are compatible with real-time BCI applications. Both the MRCP traces and their features were extracted and analyzed (Figure 3), including peak negativity, readiness potential slopes, negative slope, motor potential, and overall MRCP amplitude (PN, NS1, NS2, rebound, and PP). The preprocessing steps are described as follows (Figures 4, 5):

Line noise was removed by applying a 50 Hz notch filter using a 2nd order Butterworth filter. EEG channels were then filtered using a 2nd order Butterworth band-pass filter ranging from 0.05 to 3 Hz. Next, EEG and EMG data were segmented into epochs 4 s before to 5 s after column/row onsets (Figure 6). A column/row onset was defined as the moment the selector bar intersected the column or row containing the target letter. Offset correction was applied per epoch by demeaning – that is, by calculating the mean for each of them and subtracting it. To improve spatial resolution, a large Laplacian transformation was applied to the Cz electrode. EMG onset detection was performed using an adapted version of Yang’s algorithm, which identifies movement initiation by detecting deviations from baseline activity and image processing techniques (Yang et al., 2017). This adapted version included modifications to the threshold levels for detecting EMG activity and adjustments to the window length for morphological image processing, such as opening and closing techniques. The window duration was set to 1.5 s, based on the participants’ intent to sustain movement for approximately 2 s. This method was used solely for confirming movement execution and not for training or classifying MRCPs in real-time.

Data analysis focused on the Cz electrode and the Laplacian result of this electrode for each trial. Column/row onsets were used to segment the data and define individual trials. The EMG signal was then analyzed to confirm that the subject performed the intended movement within the appropriate time window. Both segmentation and movement verification were performed using a custom MATLAB graphical user interface (GUI).

After confirming the movement, the segmented data (trials) were inspected with the GUI to verify the presence of MRCPs. The GUI was also used to identify the Peak Negativity (PN) within each trial. A trial was considered successful, indicating the presence of an MRCP, if it met all the following criteria: (1) PN and EMG onset occurred within the time window of −0.5–0.5 s relative to the column/row onset; (2) the MRCP exhibited two distinct negative slopes followed by a rebound phase; (3) PN corresponded to a local minimum in the MRCP; or (4) PN occurred more than 1 s before the column/row onset (Figure 3).

This stepwise approach ensured that data segmentation, movement verification, MRCP validation, and PN identification were carried out systematically, resulting in the reliable selection of valid MRCPs across all trials.

The number of successful trials per phrase, condition, and subject was calculated as a percentage of the total trials (*n* = 26). In this study, this metric is referred to as the success rate, which indicates the number of times a subject successfully elicited an MRCP, as determined based on the inclusion criteria outlined in the previous paragraph. Success rates were calculated separately for both Cz and Laplacian-processed data. To standardize onset, three datasets per phrase were created, with MRCPs aligned based on three different time points: the column/row onset, the EMG onset (defined as the start of EMG activity), and the Peak Negativity (PN) timing. Each dataset was centered around the corresponding time point (column/row, EMG, or PN) and consisted of epochs extending 2 s before and 1.5 s after the specified time point. The PN-shifted dataset was selected for further analysis due to phase cancelation observed in other shifts.

Grand averages were computed per condition by grouping selected trials by condition, while Statistical Parametric Mapping (SPM) was conducted by dividing selected trials by subject and condition. For each subject-condition combination, a mean was calculated, yielding 39 average MRCPs (13 subjects * 3 conditions). These were analyzed using one-way ANOVA in MATLAB’s SPM toolbox (Pataky, 2022), with *post-hoc* paired *t*-tests and Bonferroni correction if significant results (*p* < 0.05) were found.

Descriptive features (PN, NS1, NS2, rebound, PP) were extracted (Figure 3), per trial and condition. Finally, statistical analysis involved computing the mean of each feature per subject and condition. Organized arrays were created with columns representing conditions and rows representing subjects. Five arrays, each representing a feature from both preprocessing methods (non-Laplacian and Laplacian), were analyzed using Friedman’s test. If significance was detected, a Wilcoxon signed-rank *post-hoc* test with Bonferroni correction was applied for the feature.

## 3 Results

**Figure 7:**
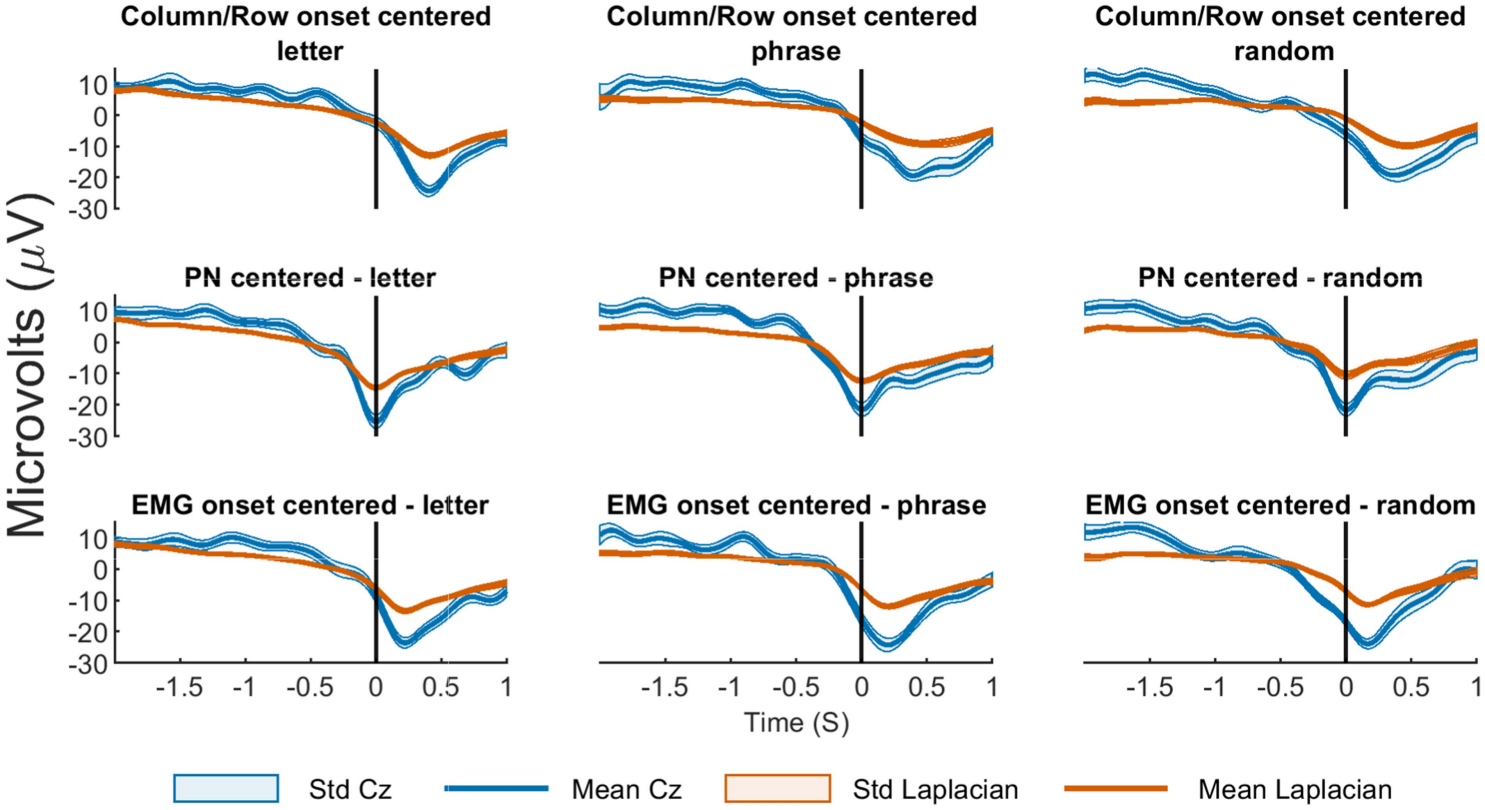
Laplacian vs. non-Laplacian on different onsets shifts. The means MRCP for subject 03 across conditions and shifting methods. The figure illustrates the mean and standard deviation of all the MRCPs, with (orange) and without (blue) Laplacian, considering shifting and condition. The graphs are organized in columns and rows. Columns represent different conditions: letter (left), phrase (middle), random (right). Rows indicate various shifting methods: no shifting or column/row (top), based on the PN (middle) and based on the EMG (bottom).

In order to identify the MRCPs, the continuous data was segmented according to the expected column/row onsets. Subsequently, two onset-shifting techniques, PN shifting and EMG onset shifting, were applied. For each subject and condition, the mean MRCP per subject across conditions and shifting methods were computed (Figure 7), to determine the best onset for feature extraction. The analysis concluded that PN shifting provided better results, making it the chosen reference point for both non-Laplacian and Laplacian data.

To evaluate whether MRCP elicitation can be effectively used with the speller, the success rates of participants were calculated. Results are presented for each subject (S01–S15, excluding S10 and S11), detailing the number of elicited MRCPs and corresponding success rates across different conditions. This analysis was conducted both without and with Laplacian filtering, the results are shown in Table 3, 4 correspondingly.

**Table 3:** MRCP success rate without Laplacian filtering.

ID subjects	Total number of elicited MRCPs			Success rate		
	Single repeated character	Spelling phrase	Random characters	Single repeated character	Spelling phrase	Random characters
S01	22	24	25	85%	92%	96%
S02	19	19	17	73%	73%	65%
S03	25	25	24	96%	96%	92%
S04	18	19	22	69%	73%	85%
S05	13	16	21	50%	62%	81%
S06	14	18	14	54%	69%	54%
S07	18	15	14	69%	58%	54%
S08	23	18	21	88%	69%	81%
S09	22	19	18	85%	73%	69%
S12	13	18	10	50%	69%	38%
S13	13	18	15	50%	69%	58%
S14	18	13	11	69%	50%	42%
S15	18	12	16	69%	46%	62%
MEAN	17.30	17.31	16.26	67%	67%	63%
SD	4.06	3.67	4.79	16%	14%	18%

The success rate, defined as the presence of an MRCP, was determined based on exclusion criteria (methods section).

**Table 4:** MRCP success rate with Laplacian filtering.

ID subjects	Total number of elicited MRCPs			Success rate		
	Single repeated character	Spelling phrase	Random characters	Single repeated character	Spelling phrase	Random characters
S01	22	25	19	85%	96%	73%
S02	19	20	18	73%	77%	69%
S03	25	25	26	96%	96%	100%
S04	17	14	15	65%	54%	58%
S05	21	19	16	81%	73%	62%
S06	17	15	17	65%	58%	65%
S07	16	16	9	62%	62%	35%
S08	17	14	18	65%	54%	69%
S09	16	16	11	62%	62%	42%
S12	12	17	14	46%	65%	54%
S13	19	17	1|	73%	65%	42%
S14	16	17	7	62%	65%	27%
S15	16	17	8	62%	65%	69%
MEAN	17.38	17.28	13.64	67%	67%	52%
SD	3.30	3.60	5.01	13%	14%	19%

The success rate, defined as the presence of an MRCP, was determined based on exclusion criteria (methods section).

For the analysis without Laplacian filtering, the mean number of elicited MRCPs of the control condition and the spelling phrase was similar (17.3 ± 4.06 and 17.31 ± 3.67) and were not significantly different (Table 3). In contrast, a slight decrease in the mean was observed for the random characters condition (16.26 ± 4.79). Success rates equal or below 50% occurred only six times for various conditions and subjects. On the other hand, 11 times different subjects, and across various conditions, achieved percentages equal to or higher than 80%.

Implementing Laplacian filtering did not significantly improve the mean success rate (Table 4) of the control condition or the spelling phrase (17.38 ± 3.30 and 17.28 ± 3.60). Similarly, in the processing without Laplacian, these means did not exhibit significant differences between the two conditions. The most notable difference was observed in the random characters condition, where the mean decreased to almost 50% success rate (13.64 ± 5.01).

The implementation of the Laplacian filter enhanced or matched the success rate to the non-Laplacian results, for six subjects in at least two out of the three conditions-specifically, S01, S02, S03, S05, S06, and S15.

Additionally, there was a consistent or increased success rate observed from the spelling phrase condition compared to the control condition in eight participants:

- Non-Laplacian: S01, S02, S03, S04, S05, S06, S12, S13.
- Laplacian: S01, S02, S03, S07, S09, S12, S14, S15.

In contrast, when comparing the control condition to the random one, a decrease in success rate was noted for eight subjects in both the non-Laplacian and the Laplacian results.

- Non-Laplacian: S02, S03, S07, S08, S12, S13, S14, S15.
- Laplacian: S01, S02, S04, S05, S07, S09, S13, S14.

### 3.1 Feature—statistical analysis

To assess whether the interface and spelling task affect the morphology of the MRCPs, features were extracted from all selected trials for each subject and condition. The mean of each feature was computed for each subject and condition, and repeated for all features. These means were then statistically compared for both processing methods and the results are shown in Table 5 (non-Laplacian) and Table 6 (Laplacian).

**Table 5:** Friedman test results for the non-Laplacian data.

NS1	NS2	PP	PN	Rebound
0.1988	0.116	0.5836	0.7939	0.3679

**Table 6:** Friedman test results for the Laplacian data.

NS1	NS2	PP	PN	Rebound
0.926	0.3973	0.0231*	0.116	0.2319

The asterisk (*) indicates that the value is statistically significant, with *p*-values less to 0.05.

From the Friedman test results, only one feature showed significance (*p* < 0.05). This feature was the PP in the Laplacian dataset (*p* = 0.0231). A Wilcoxon rank-sum *post-hoc* test was performed for pairwise comparisons with a Bonferroni correction (adjusted significance level: *p* < 0.0167), with results presented in Table 7. No significant differences were found in this test. The comparisons of the features between the control condition and the phrase (*p* = 0.1272) and against the random condition (*p* = 0.0574) were the smallest, but neither showed significant differences.

**Table 7:** *Post-hoc* Wilcoxon rank sum test results for PP feature from Laplacian data.

Control vs. phrase spelling	Control vs. random	Phrase vs. random
0.1272	0.574	0.5417

### 3.2 MRCP template—SPM

**Figure 8:**
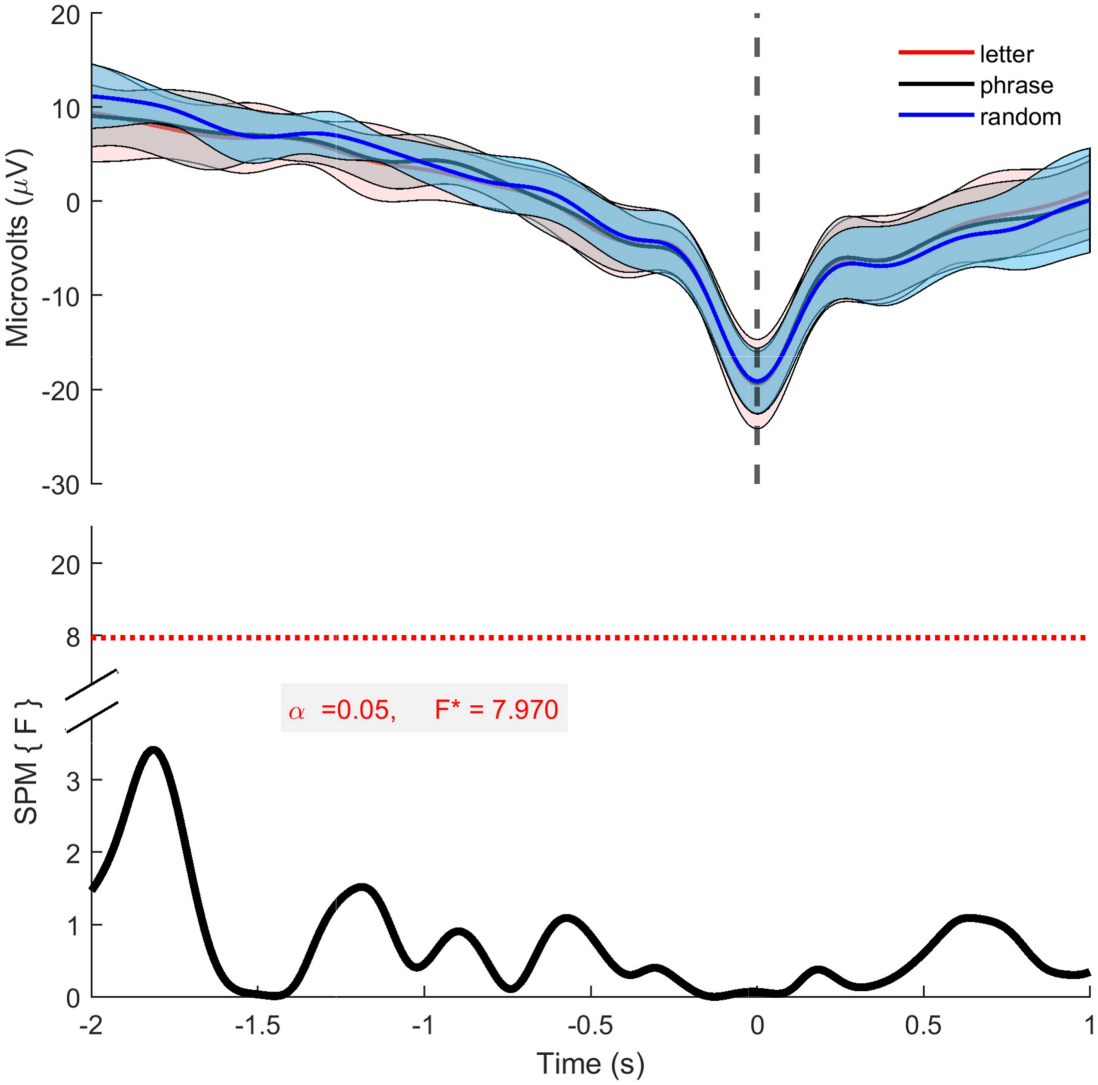
Cz grand average MRCP data. The top panel displays the grand averages of the 12 participants (trials mean of 17.30—letter, 17.31—phrase 16.24 -random) for each condition, the dashed line over the y axis represents the PN of the grand averages. The lower trace presents the SPM analysis results. Both indicate that there are no differences across the three conditions.

To compare the three conditions, we calculated the grand average of the MRCPs for each condition and conducted an SPM analysis. The grand averages were obtained by averaging the individual MRCP traces over three conditions (Figure 8). The resulting grand averages across conditions exhibit similar morphology, with overlapping confidence intervals.

To further investigate these similarities, an SPM analysis was conducted using a one-way ANOVA. For this analysis, MRCPs were first averaged per subject for each condition, resulting in 39 averaged MRCPs (13 subjects * 3 conditions). The SPM analysis confirmed that there were no statistically significant differences between the three conditions, consistent with the observed grand averages.

**Figure 9:**
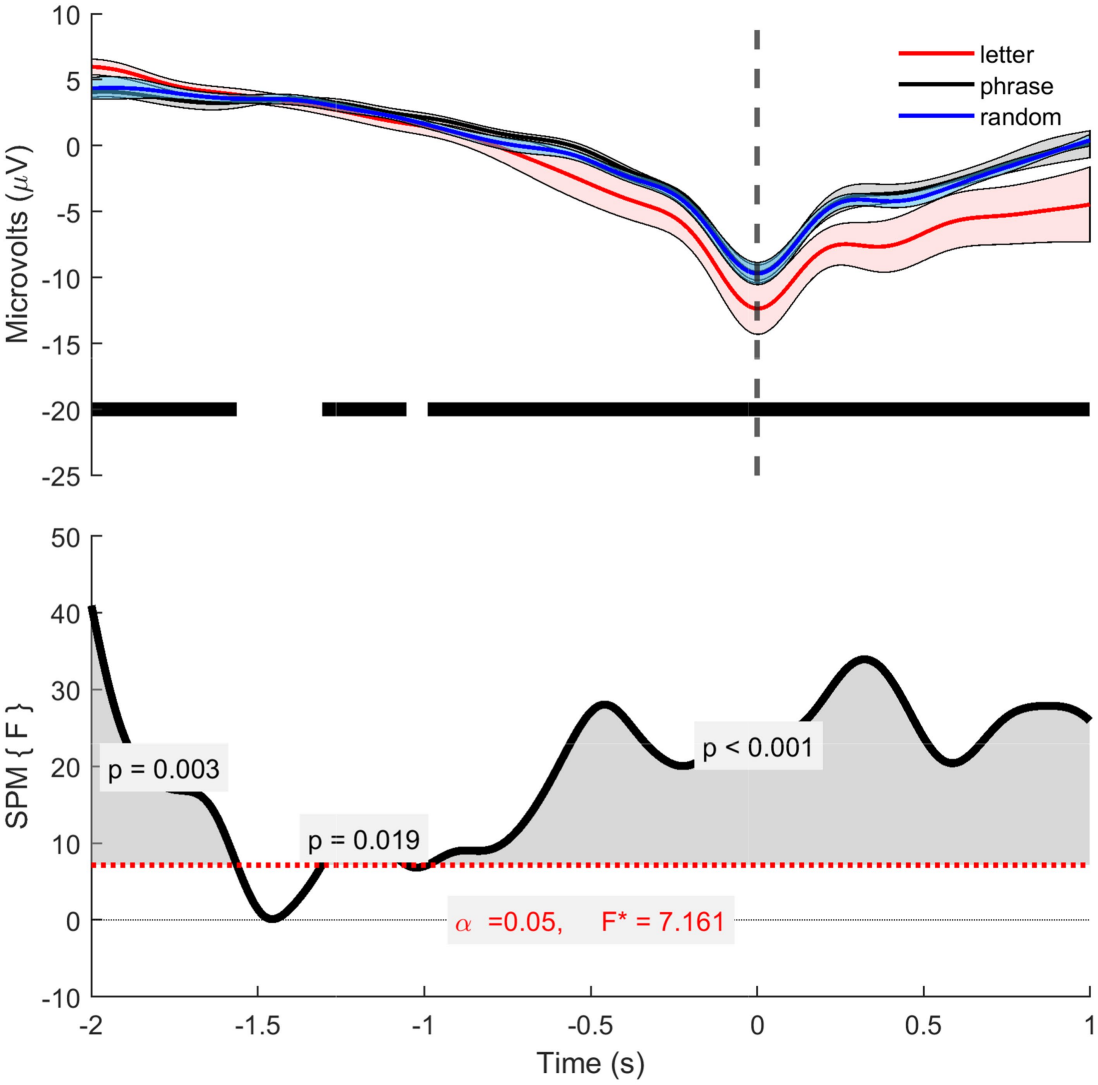
The Laplacian data. The top panel displays the grand averages of the 12 participants (trials mean of 17.38—letter, 17.28—phrase 13.64 -random) for each condition, the dashed line over the y axis represents the PN of the grand averages, while the black traces show the times where there was significance between conditions. The lower trace presents the SPM analysis results, where the grey shaded area represents the significance.

The Laplacian data showed significant differences across conditions except between −1.56 s and −1.3 s (Figure 9). *Post-hoc* paired *t*-tests with Bonferroni correction (Supplementary Figure 1) revealed the following: For the control vs. phrase condition, only the interval from −1.55 s to −1.0 s was not significant (*p* ≤ 0.2). For the letter vs. random condition, significant differences were found between −2.0 s and −1.88 s, −0.53 s and −0.36 s, and −0.17 s and 1.5 s, with the letter showing the greatest difference. The phrase vs. random condition had a significant difference only between −0.94 s and −0.4 s (p ≤ 0.2).

## 4 Discussion

The aim of the current study was to assess the feasibility of using MRCPs as a control signal for a BCI-speller system. Specifically, we focused on analyzing the MRCP and its features under the cognitive load imposed by the simulation of a spelling task generated by the BCI speller that was designed for this purpose. Our findings indicate that participants successfully generated MRCPs during the tasks, and no significant differences were observed across conditions for the MRCP features. The SPM analysis revealed some differences between conditions only for the Laplacian channel/data. In the following subsections we will provide a detailed discussion of the results.

### 4.1 MRCP during the spelling tasks

In the field of BCI, selecting an appropriate control signal that accurately reflects the user’s intent is critical for the system’s effectiveness (Abiri et al., 2019; Krusienski et al., 2012; Wolpaw et al., 2002). In this study, we chose MRCPs as a potential control signal for BCI spellers.

We implemented the success rate, defined as the occurrence of an MRCP, as a metric to evaluate the correlation between the user’s intent and the actual brain output measured by EEG. High success rates suggest that the MRCP has the potential to serve as a reliable control signal, capable of effectively capturing the user’s intent.

Compared to the control condition, the success rates of the phrase spelling condition met expectations, with 9 out of 12 subjects achieving at least 69% success. Notably, six participants were novices, suggesting that MRCPs can be elicited without extensive user training (Jochumsen et al., 2018; Niazi et al., 2013). This trend persisted in the phrase spelling condition, where 10 participants matched or exceeded their performance, supporting the idea that MRCP-based control can be trainable (Jochumsen et al., 2017). While this may appear lower than classification accuracies reported in P300 and SSVEP studies (Abiri et al., 2019; Rezeika et al., 2018), it is important to note that participants in this study had no prior experience or training in eliciting MRCPs. Despite this lack of user training, MRCPs were successfully elicited, suggesting that performance could improve with additional practice and adaptation.

On the other hand, the random condition revealed that just seven subjects achieved an equal or higher success rate than 69%. This decrease could be attributed to the diversion of attention (Aliakbaryhosseinabadi et al., 2017a; Aliakbaryhosseinabadi et al., 2017b), or the complexity of selecting random characters due to the lack of goal-oriented spelling (Frömer et al., 2012; Olsen et al., 2021).

The Laplacian filter improved or maintained the success rate in six subjects across at least two of three conditions but decreased in the others. As a spatial filter, the Laplacian reduces spatial noise by emphasizing local signal sources and minimizing distant ones. While effective in subjects with well-localized MRCPs, the filter can introduce distortions in cases where signals are more diffuse. Additionally, the Laplacian has been shown to counteract attention diversion during tasks, potentially explaining its improved performance in some subjects (Aliakbaryhosseinabadi et al., 2017a; McFarland, 2015).

### 4.2 MRCP template

The grand average MRCPs were computed based on three different onsets (column/ row, PN and EMG), revealing a phase-canceling effect for the column/row and EMG onset. The PN serves as the crucial point marking the end of the negative shift and the beginning of the positive shift (Do Nascimento et al., 2006; Shibasaki and Hallett, 2006). Therefore, it proves to be an excellent reference point for extracting the MRCP features with consistency. Additionally, this selection aligns with the goal of enabling non-muscular control signals as it remains present during motor imagery (Cunnington et al., 1996), which are essential for BCI applications, especially in patients with motor impairments.

Once an onset was selected the MRCPs were analyzed with and without Laplacian filtering. In the non-Laplacian data, MRCPs behaved as expected, showing no statistically significant differences across conditions, either in feature extraction or SPM analysis. Prior research indicates that MRCP morphology, particularly in features like NS1 and NS2, can change with attention diversion (Aliakbaryhosseinabadi et al., 2017a; Aliakbaryhosseinabadi et al., 2017b; Wright et al., 2012). The absence of significant changes here suggests that the spelling task did not impose a cognitive load that interfered with the movement timing or focus.

The use of the Laplacian filter in processing MRCP signals presents both benefits and challenges, particularly when comparing tasks with different cognitive demands. During spelling tasks, which are likely to engage broader cortical regions due to the increased complexity, the Laplacian filter may show amplitude differences in the MRCP. This occurs because the filter enhances localized neural activity but may smooth out signals spread across multiple channels, which is more common in cognitively demanding tasks. In contrast, monotonic tasks such as the control condition, are less affected by the Laplacian because they involve more focused neural activity that tends to produce clearer MRCP signals (Aliakbaryhosseinabadi et al., 2017a; Aliakbaryhosseinabadi et al., 2017b; McFarland, 2015).

Interestingly, in our analysis, while no significant differences were found in the extracted MRCP features, the SPM analysis of the Laplacian-processed data did show differences across conditions. This suggests that the Laplacian filter, while effective at reducing noise, might amplify specific aspects of the MRCPs and reduce their overall amplitude. This suggests that while Laplacian filtering can refine the signal by reducing noise, it may also alter the MRCP morphology, warranting careful consideration in future studies.

### 4.3 Comparison of MRCP-based BCIs with existing paradigms

P300 and SSVEP spellers have established themselves as reliable BCI paradigms, consistently achieving accuracy rates of 80–95% with information transfer rates of 20–25 bits/min (Gannouni et al., 2022; Kundu and Ari, 2022; Rezeika et al., 2018). However, they require multiple stimulus presentations for each selection and can cause visual fatigue during extended use (Abiri et al., 2019; Gannouni et al., 2022; Rezeika et al., 2018).

In contrast, MRCP-based BCIs offer single-trial detection capability with accuracy rates of 75–85%, though their inherent low-frequency characteristics (~2 s per decision) may result in lower information transfer rates depending on the temporal analysis window (Jochumsen et al., 2020; Olsen et al., 2021; Savić et al., 2021). Recent advances in MRCP detection, including predictive algorithms for low-latency detection and hybrid approaches combining MRCP with ERD/ERS features to increase detection accuracy, show promise in reducing these speed and accuracy limitations (Savić et al., 2014; Savić et al., 2020).

MRCPs offer several advantages for BCI control, including reduced dependence on external stimulation, real-time feedback, and short latency, which could enhance usability and effectiveness in real-world applications (Savić et al., 2020, 2021). However, unlike SSVEP- and P300-based BCIs, which achieve high classification accuracy with minimal user training, MRCP-based systems may require further refinement and validation to reach comparable reliability. Although this study did not implement an online algorithm or automated classification, previous research has demonstrated the successful integration of MRCPs into BCI systems, highlighting their potential as a viable control signal (Savić et al., 2021).

### 4.4 Limitations

While this study offers promising insights into the development of an MRCP-based BCI speller, several limitations should be considered for future research. Participants performed the spelling task without any prior training. Additional training could improve the reliability of MRCPs elicitation, as some participants exhibited increased success after just one session (Jochumsen et al., 2017). Furthermore, the experimental sessions lasted only 5–10 min, which does not reflect the real-world use of a BCI speller. Investigating MRCP viability for longer periods, accounting for fatigue, is essential for practical applications.

This study demonstrated that MRCPs can be elicited in BCI-naïve users during a simulated BCI-speller task. However, a crucial limitation is that the analyzed MRCPs were derived from *executed* movements, not imagined or attempted ones. This may constrain the interpretation of our results, as they primarily reflect the feasibility of MRCP-based BCIs in scenarios where voluntary motor execution is possible. While MRCPs can be elicited by both executed movements and motor imagery (Do Nascimento et al., 2006; Gu et al., 2013; Shakeel et al., 2015), MRCPs elicited during imagined movements are often weaker and exhibit different characteristics (Do Nascimento et al., 2006; Gu et al., 2013). However not so much is known with regards to attempted movement – thus where patients unable to move due to paralysis attempt to perform the task. Previous research has demonstrated the feasibility of using MRCPs elicited by motor intention (i.e., motor imagery) (Aliakbaryhosseinabadi et al., 2020; Jiang et al., 2015; Niazi et al., 2013; Savić et al., 2021), however, the inherent differences in MRCP characteristics between executed and imagined movements, particularly the potentially weaker signal strength during motor imagery, are critical considerations for future development of MRCP-based BCIs for communication and control. Future research must validate the applicability of this approach in individuals with limited motor abilities.

Even though MRCPs have the potential to be used in BCIs without external stimulation, the specific implementation in this study used a visual speller interface with moving selectors to guide letter selection. This visual interface, while different from flashing stimuli, could also potentially contribute to visual fatigue during extended use, although this was not directly assessed in the present study.

In this study, the presence of the MRCPs was determined through manual inspection of the EEG data using exclusion criteria predefined by the researchers. Each trial was evaluated based on these criteria, and only trials meeting the specified conditions were counted toward the success rate. This systematic approach ensured consistency in MRCP identification. To advance the practical application of MRCP-based BCIs, future work will focus on developing and implementing machine learning algorithms for automated MRCP detection and classification, which could improve accuracy and efficiency in both executed and imagined movement scenarios.

Additionally, while high-pass filters have been used to reduce eye and head movement artifacts, MRCPs are in the frequency range of these artifacts meaning the filters may not be fully effective. Future research should integrate eye tracking and head movement measurements to improve signal quality. The current study was conducted offline, and the next step is to perform online trials to assess real-time performance. In an online setting, MRCPs may be influenced by factors like fatigue, feedback and user errors, making another metrics such as false positives, precision, and accuracy crucial for evaluating system effectiveness.

## 5 Conclusion

Significant progress has been made in non-invasive BCI spellers using P300, Steady-State Visual Evoked Potentials (SSVEP), and Motor Imagery (MI) paradigms, each offering advantages such as rapid response (P300), high information transfer rates (SSVEP), and well-established control schemes (MI induced ERSPs) (Cecotti, 2011; Rezeika et al., 2018). However, these approaches also face challenges including reliance on external stimulation (P300, SSVEP), which can lead to fatigue, and extensive user training requirements (MI) (Abiri et al., 2019; Kundu and Ari, 2022; Nicolas-Alonso and Gomez-Gil, 2012; Rezeika et al., 2018). Here we analyzed the potential of MRCPs as an alternative approach, focusing on their elicitation during a simulated spelling task.

This study explored the feasibility of MRCPs as an alternative control signal for BCI spellers, focusing on their elicitation during a simulated spelling task with untrained participants. While potential advantages of MRCP-based BCIs have been suggested in prior research (Aliakbaryhosseinabadi et al., 2020; Aliakbaryhosseinabadi et al., 2017a; Aliakbaryhosseinabadi et al., 2017b; Savić et al., 2021) our study did not directly assess them but demonstrated that MRCPs can be reliably elicited with motor execution in an offline BCI-spelling simulation.

The Laplacian filter’s impact was mixed—enhancing performance in some cases while reducing it in others—yet MRCPs consistently proved to be a promising and practical alternative for BCI spellers, which may enhance their usability and effectiveness in specific users and application scenarios. These findings suggest that MRCPs could address some of the limitations of existing paradigms, providing a feasible solution for future BCI applications.

While our findings provide an initial assessment of MRCPs for BCI spellers, direct generalization to populations with neurological conditions like ALS requires further investigation. This will be an area of focus in our upcoming research.

## Data Availability

The raw data supporting the conclusions of this article will be made available by the authors, without undue reservation.

## Supplementary material

Supplementary Figure 1 The Laplacian data - *post-hoc* tests. The figure shows the grand averages of the compared conditions and the corresponding *post-hoc* tests with a Bonferroni correction. The left trace shows the comparison between the control and the phrase condition. The middle trace shows the comparison between the letter and the random condition. The right trace shows the phrase vs. the random condition. The black lines on the top plots indicate where in time there was significance between the conditions. The grey shaded areas in the bottom plots represent the significance.
