# Supplementary figures and images for: Toward brain-computer interface speller with movement-related cortical potentials as control signals
Source: Front Hum Neurosci. 2025 Apr 2;19:1539081. doi: 10.3389/fnhum.2025.1539081 (PMC11999959; doi:10.3389/fnhum.2025.1539081)

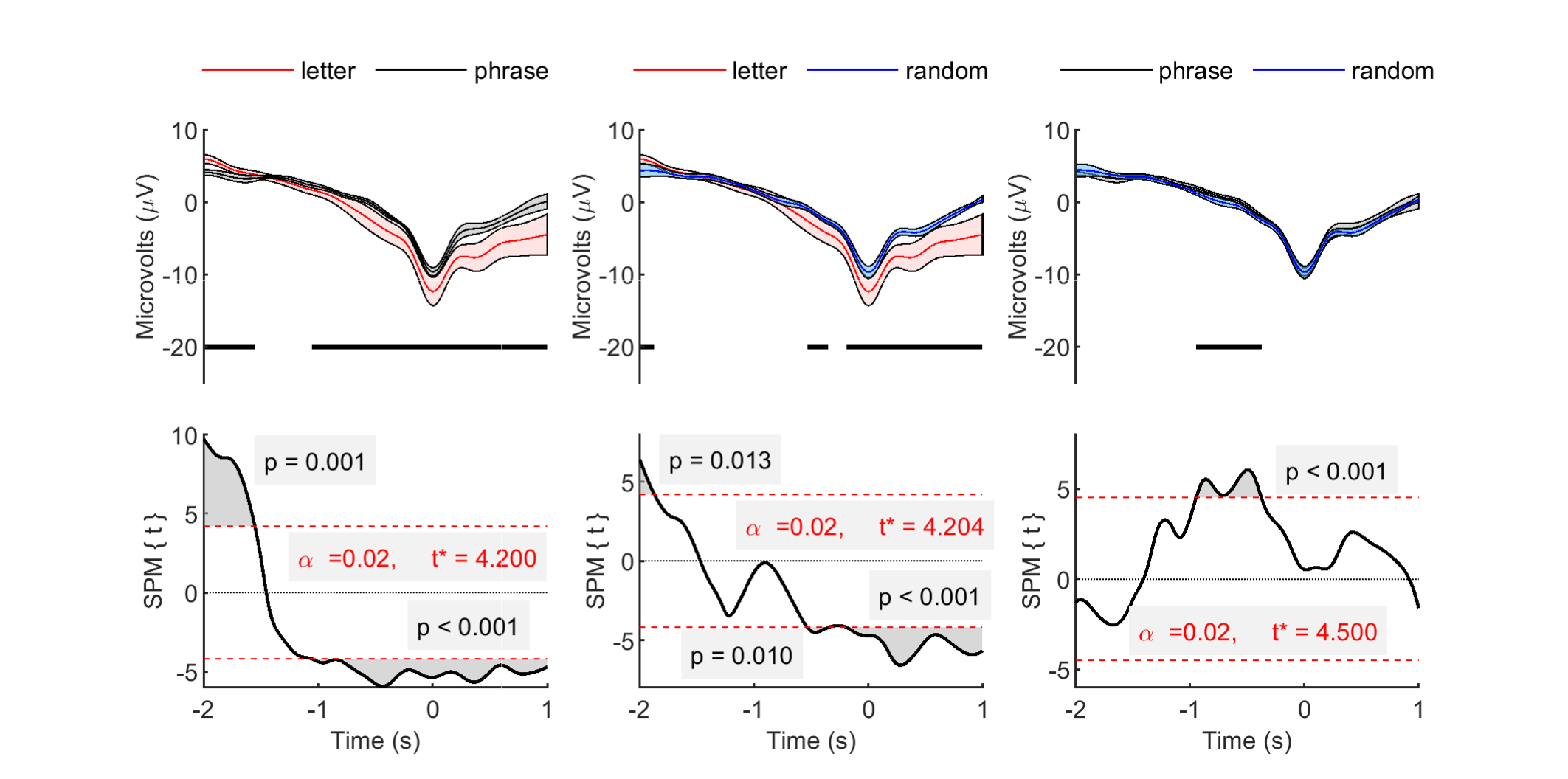

Supplement: Supplementary Figure 1 — The Laplacian data - post-hoc tests. The figure shows the grand averages of the compared conditions and the corresponding post-hoc tests with a Bonferroni correction. The left trace shows the comparison between the control and the phrase condition. The middle trace shows the comparison between the letter and the random condition. The right trace shows the phrase vs. the random condition. The black lines on the top plots indicate where in time there was significance between the conditions. The grey shaded areas in the bottom plots represent the significance. [file Image_1.TIFF]
